# Short chain fatty acids and colon motility in a mouse model of irritable bowel syndrome

**DOI:** 10.1186/s12876-021-01613-y

**Published:** 2021-01-26

**Authors:** Ilnar F. Shaidullov, Dina M. Sorokina, Farit G. Sitdikov, Anton Hermann, Sayar R. Abdulkhakov, Guzel F. Sitdikova

**Affiliations:** 1 Institute of Fundamental Medicine and Biology, Kazan Federal University, 18, Kremlevskaya str., 420008 Kazan, Russia; 2 Department of Biosciences, University of Salzburg, Hellbrunnerstr.34, 5020 Salzburg, Austria

**Keywords:** Irritable bowel syndrome (IBS), Short chain fatty acids (SCFAs), Mouse colon motility, Colonic transit, Sodium propionate, Sodium  acetate, Butyric  acid

## Abstract

**Background:**

Irritable bowel syndrome (IBS) is defined as a multifactorial disorder associated with visceral hypersensitivity, altered gut motility and dysfunction of the brain-gut axis. Gut microbiota and its metabolites are proposed as possible etiological factors of IBS. Short chain fatty acids (SCFAs) induce both inhibitory and stimulatory action on colon motility, however, their effects on the IBS model were not investigated. The aim of our study was to investigate the level of SFCAs in feces and their effects on colon motility in a mouse model of IBS.

**Methods:**

IBS model was induced in mice by intracolonic infusion of 1% acetic acid during the early postnatal period. Mice colon hypersensitivity was assessed by the threshold of the abdominal withdrawal reflex in response to colorectal distention. Colon contractility was studied using proximal colon specimens in isometric conditions. Transit rates were assessed by the pellet propulsion in the isolated colon. Concentrations of SCFAs in feces were measured using gas–liquid chromatography.

**Results:**

The concentration of SCFAs in feces of IBS model mice was higher compared to the control group. Visceral sensitivity to colorectal distension and colonic transit rate were increased indicating IBS with predominant diarrhea. The frequency and amplitude of spontaneous contractions of proximal colon segments from IBS mice were higher, but carbachol induced contractions were lower compared to control. During acute application of SCFAs (sodium propionate, sodium acetate or butyric acid) dose-dependently (0.5–30 mM) decreased tonic tension, frequency and amplitude of spontaneous and carbachol-evoked contractions. In the mouse IBS group the inhibitory effects SCFAs on spontaneous and carbachol-evoked contractions were less pronounced. At the same time intraluminal administration of butyrate (5 mM) increased the transit rate in the colon of both groups, but its stimulatory effect was more pronounced in mouse IBS model group.

**Conclusion:**

Our data indicate that the increased transit rate in the mouse IBS model group is associated with a disbalance of activating and inhibiting action of SCFAs due to chronically elevated SCFA levels, which may impact the pathogenesis of IBS with predominant diarrhea syndrome.

## Background

The irritable bowel syndrome (IBS) is a functional gastrointestinal disorder defined by a variable combination of chronic or recurrent gastrointestinal symptoms including abdominal pain and changed bowel habits as a result of altered intestinal motility, dysfunction of the brain-gut axis and visceral hypersensitivity. IBS affects up to 20% of the entire world population and has not been able so far to be explained by morphological or biochemical abnormalities [1, 2]. In accordance with Rome IV criteria, IBS can be classified according to the prevailing clinical symptoms into four subtypes: IBS with predominant constipation (IBS-C), IBS with predominant diarrhea (IBS-D), IBS with mixed bowel habits (IBS-M) and unclassified IBS [3]. Despite its high prevalence, the etiology and pathophysiology of IBS remains poorly understood and is often referred to as multifactorial disease [4, 5].

Recently, the role of intestinal microbiota in the pathogenesis of IBS has become apparent, although it is not clear whether it is a cause or consequence of IBS [6]. It is known that microbiota can affect the intestinal motility, the integrity of the intestinal barrier, local immune responses and nervous regulation [7]. Short-chain fatty acids (SCFAs), including acetate, propionate and butyrate are fermentation products of carbohydrates and occur in molar ratio of 3:1:1 in the colon [8]. The main SCFA-producing bacteria in the gut are obligate anaerobes [9]. Culture-based and molecular studies discovered that IBS is accompanied by a lesser extent of diversity of microbial populations and altered proportion of bacterial groups which included decreased levels of fecal lactobacilli and bifidobacteria, increased levels of facultative anaerobic bacteria dominated by streptococci and *Escherichia coli* (*E. coli*), increased ratios of *Firmicutes*: *Bacteroidetes* and higher counts of anaerobic organisms (such as *Clostridium*) [10]. SCFAs are used by the microbiota for growth and maintenance of host cellular functions [11]. In the human colon SCFAs are accumulated in concentrations up to 150 mmol/l and represent the major organic anions [12], which are considered to play an important role in the regulation of colon motility. Effects of SCFAs are dependent on the specific SCFA, segment of the colon, animal species and experimental models [13, 14]. Inhibitory effects of SCFAs at physiological concentrations (10–30 mM) [15] were proposed to be mediated by the enteric nervous system [16] and release of the antimotility peptide YY (PYY) from enteroendocrine cells [17, 18]. At the same time in mucosa attached preparations SCFAs stimulated contractility in low concentrations (1–10 mM) [19–22] suggesting a role of paracrine or hormonal agents like serotonin released from enteroendocrine cells [13].

The concentration of SCFAs depends on the intestinal microbiota content and may impact the pathogenesis of IBS [23]. Recent meta-analysis data demonstrated differences in fecal SCFA level between healthy controls and patients with IBS, where in IBS-C patients, propionate and butyrate were reduced, whereas butyrate was increased in IBS-D patients [24]. Although IBS is characterized by intestinal motility dysfunction [25, 26], the underlying mechanisms are not clear. The aim of our study was to analyze the feces level of SCFAs and the effects of SCFAs on the contractility of the isolated proximal colon specimen and colon transit rate in a mouse model of IBS.

## Methods

### Animals

Experiments were performed using male BALB/c mice bred and maintained in an animal housing facility at Kazan Federal University. All experiments were performed in accordance with the European Directive 86/609/EEC of 24 November 1986 and approved by the Local Ethics Committee at the Kazan Federal University (protocol No.8 issued May 5, 2015). Mice were housed individually and fed food and water ad libitum under controlled environmental conditions at 21 ± 2 °C in a light–dark room. All neonates used in the experiments were housed per cage together with one adult female mouse until they were one-month-old.

### Induction of irritable bowel syndrome

The model of post-inflammatory irritable bowel syndrome (IBS), an experimental model of neonatal sensitization of mice, was induced by rectal administration of 1% acetic acid solution [27], which causes visceral hypersensitivity in adult animals without signs of histological inflammation [28]. 47 animals were used in the experiments. Animals were randomly divided into two groups—IBS model (n = 21) and control group (n = 26). In the IBS model group animals were subjected to acetic acid infusion for 10 days beginning from postnatal day (P) 10 (0.3 ml daily for P10-P15 and 0.5 ml daily for P16-P21). Experiments to determine visceral hypersensitivity and colon motility were performed two weeks after the last injection of acetic acid solution.

### Evaluation of visceral sensitivity

Mice colon hypersensitivity was assessed by measuring the threshold intensity of the abdominal withdrawal reflex (AWR) arising in response to colorectal distention [29]. Distention was applied using an arterial embolectomy catheter (4F-Fogarty, Edwards Lifesciences LLC, Irvine, CA, USA) which was inserted rectally into the descending colon of anesthetized mice (2% isoflurane) and fixed at the base of the tail. AWR measurements were carried out 30 min after wake-up and reorientation of the animals. Furthermore, visual observation of the reaction to the rapid phase stretching of the balloon for 20 s in ascending order (0.1, 0.25, 0.35, 0.5, and 0.65 ml) was carried out. The response of the animal to colorectal distention was assessed on an AWR scale: 0, no behavioral response; 1, brief movement of the head, followed by immobility; 2, contraction of abdominal muscles; 3, lifting the abdomen, and 4, body flexion and pelvic lift [28]. Each measurement was repeated three times with 30 s intervals.

### Recording of contractile activity of proximal colon specimen

The colon was removed from adult mice (approx. 45–50 days old) sacrificed by cervical dislocation and segments were placed into Krebs solution containing (in mM): 121.0 NaCl, 5.9 KCl, 2.5 CaCl_2_, 1.2 MgCl_2_, 25.0 NaHCO_3_, 1.2 NaH_2_PO_4_, 8.0 C_6_H_12_O_6_; pH 7.2–7.4, bubbled with 95% O_2_ and 5% CO_2_, at 37 °C. To prevent any changes associated with the elevation of sodium ions from the high concentrations of sodium acetate or sodium propionate (10 and 30 mM) the bath solution was corrected accordingly. In addition, the pH of the solutions with SCFAs was checked and corrected to pH 7.2–7.4. The mouse colon was divided into three parts including the proximal, middle and distal colon. We used the proximal part of the large intestine, located below the cecum occupying one third of the total length of the colon. The colon segments 5–7 mm length were fixed along the mesenteric border with stainless clips and placed in an organ chamber. The contractile power of isolated colon segments was studied under isometric conditions using a 4-channel isolated organ bath system (Biopac Inc., Goleta, CA, USA) according to our previous study [30, 31]. The specimen was suspended upright in the bath (20 ml) with the upper part connected to the tensometric force transducer (TSD125C, Biopac Inc., Goleta, CA, USA) and the lower part fixed on a fastened hook. Each specimen was equilibrated before the experiment for 1 h under tension of 1 g and washed every 10 min with Krebs solution. Spontaneous or carbachol-evoked contractile activity was recorded in order to obtain control values, thereafter SCFAs were applied. Amplitude, tonic tension and frequency of spontaneous contractions were measured. The carbachol-evoked (1 μM) contractions were analyzed by assessing the maximum amplitude of the first peak and the area under the curve (AUC) during 1 min agonist application. In order to compare the effects of SCFAs in IBS model and control groups the data were normalized related to the initial values before the drugs were applied. Recording and subsequent analysis of the contraction parameters were carried out using Acqknowledge 4.1 (Biopac Inc., Goleta, CA, USA).

### Artificial pellet propulsion measurement in mouse colon in vitro

Colonic transit was assessed as artificial pellet propulsion in the mouse colon isolated from the caeco-colonic junction to the rectum [32, 33]. The colon was placed in oxygenated Krebs solution and the movement of an artificial black silicone pellet (with length 7 mm and diameter 2 mm) was monitored for 20 min [34]. Butyric acid (5 mM) was applied intraluminally at a rate of 0.5 ml/min. Pellet propulsion was assessed by taking photographs at t = 0 and t = 20 min. The colonic transit was calculated as the mean distance travelled relative to total length in % [32].

### Chemicals

Sodium salts of acetate, propionate and butyrate as well as butyric acid, carbachol, sodium chloride, potassium chloride, calcium chloride, magnesium chloride, sodium bicarbonate, potassium phosphate, and glucose were purchased from Sigma Aldrich (USA).

### Short chain fatty acid determination in feces

The fecal pellets were collected directly from mice at 45 day after birth and stored at − 80 °C prior to SCFA determination. SCFAs were quantified in feces samples (1 g) by gas–liquid chromatographic analysis (GLC; Agilent, USA) [35]. Commercial acetic, propionic and butyric acids were used as standards [35]. The overall level of SCFAs is presented as the sum of all concentrations of acids and isoacids in µg/g.

### Statistical analysis

Statistical analysis were performed using nonparametric and parametric statistics methods using the OriginPro software (OriginLab, CA, USA). EC_50_ was calculated from dose-dependence curves using sigmoidal curve-fitting (y = A1 + (A2—A1)/(1 + 10^(*Logx*0−*x*)*p*^), with A1—the minimum effect, A2—the maximum effect, *Logx*_*0*_*—*center (10^*Logx0*^ = EC_50_) and p—power of sigmoidal curve) [36]. The difference between data was assessed using Student *t*-test or ANOVA for multiple comparisons followed by Bonferroni post hoc test. The paired Student *t*-test was used to examine the effects of SCFAs in control or IBS groups. All data are presented as mean ± standard error. Differences were considered significant when *p* < 0.05; *n* indicates the number of animals.

## Results

### Visceral hypersensitivity

No differences in abdominal withdrawal reflex (AWR) between animals of the IBS model and control groups were observed at low distention volumes of 0.1 and 0.25 ml (n = 20, *p* > 0.05). At volumes of 0.35 ml and 0.5 ml the AWR was significantly higher in the IBS model group compared to the control group (Table 1). At a volume of 0.65 ml no significant differences between the groups were observed, which may be associated with a high level of stimulation and increased intensity of the response to irritation [28]. The data indicate that visceral sensitivity to colorectal distension was increased in the mouse IBS model.

**Table 1:** Abdominal withdrawal reflex scores for colon distention of mice from IBS model and control groups

Group	n	Balloon volume, ml				
		0.1	0.25	0.35	0.5	0.65
AWR score						
Control	20	0.37 ± 0.09	0.83 ± 0.19	1.83 ± 0.18	2.9 ± 0.14	3.63 ± 0.11
IBS model	15	0.53 ± 0.09	1.21 ± 0.11	2.56 ± 0.22*	3.38 ± 0.18*	3.82 ± 0.07

AWR scale: 0, no behavioral response; 1, brief movement of the head, followed by immobility; 2, contraction of abdominal muscles; 3, lifting the abdomen, and 4, body flexion and pelvic lift [25]

*p < 0.05 compared with the control group

### Contractility of mouse colon segments and transit rate

**Fig. 1:**
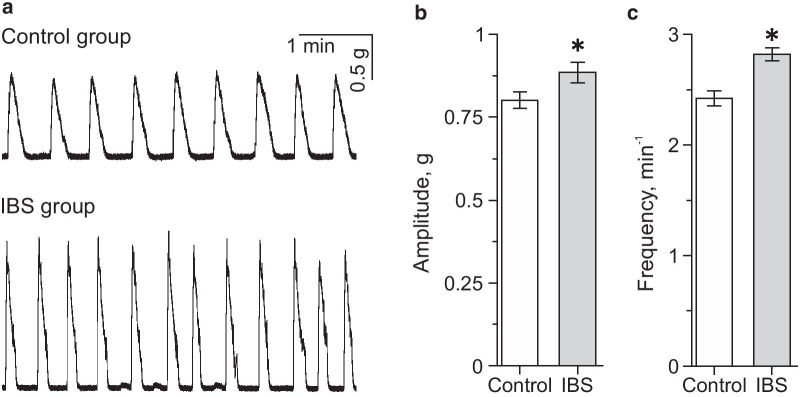
Spontaneous contractile activity of proximal colon from control and mouse IBS model groups. **a** Original recordings of spontaneous colon specimen activity in control and IBS model groups. Average amplitude (**b**) and frequency (**c**) of spontaneous contractions of specimen  from  the control (white column) and IBS model (grey column) groups. *p < 0.05 compared to the control group

To study the effect of SCFAs on gastrointestinal contractility, we analyzed parameters of contractile activity of proximal colon segments which demonstrated spontaneous activity starting about 45 min after mounting the specimen (Fig. 1a). In the IBS model group (n = 15) a larger amplitude—0.88 ± 0.02 g (*p* < 0.05) and higher frequency of contractions—2.82 ± 0.06 min^−1^ (*p* < 0.05) were observed (Fig. 1a–c). In the control group, the average frequency of spontaneous contractions was 2.42 ± 0.07 min^−1^ and the average amplitude—0.81 ± 0.02 g (n = 20) (Fig. 1b, c).

**Fig. 2:**
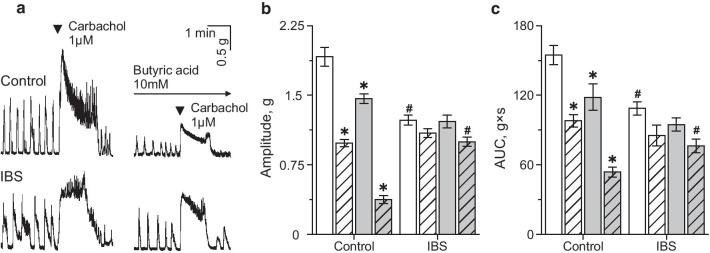
Effects of SCFAs on carbachol-evoked contractions of isolated mouse proximal column. **a** Original traces of carbachol-evoked contractions of proximal column before and after butyric acid (10 mM) incubation in control and in IBS model groups. Amplitude (**b**) and area under curve (AUC) (**c**) of carbachol-evoked contractions before (white column) and after incubation in sodium acetate (10 mM, dashed column), sodium propionate (10 mM, grey column) and butyric acid (10 mM, grey dashed column) in the control and IBS model groups. *p < 0.05 compared to carbachol effect before SCFAs application; ^#^*p* < 0.05 compared to carbachol-evoked contractions in the control group

1 μM carbachol, a non-specific muscarinic cholinergic receptor agonist during one minute application caused a contraction of the proximal colon specimen with a sharp increase of the amplitude followed by a slow decay of tension (Fig. 2a). The average amplitude of the initial increase was significantly lower in the IBS model group—1.23 ± 0.05 g (n = 15) than in the control group—1.91 ± 0.11 g; n = 20, *p* < 0.05; Fig. 2a, b). The area under the curve (AUC) was also smaller in IBS mice (108.62 ± 5.83 g × s, n = 15), compared to the control group (154.78 ± 8.22 g × s; n = 20, *p* < 0.05; Fig. 2c).

**Fig. 3:**
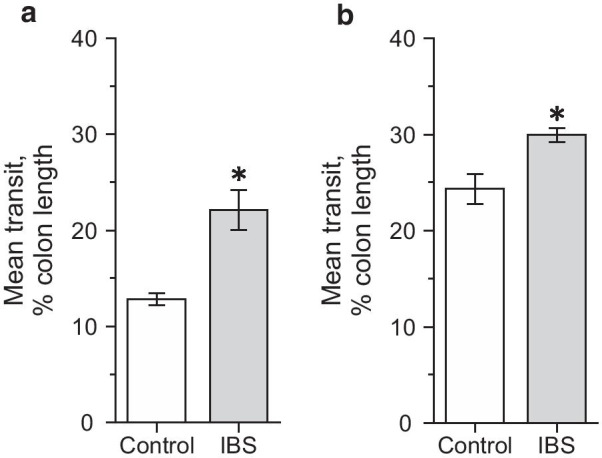
Colonic transit in control and mouse IBS model groups. Artificial pellet transit in colon of control (white column) and mouse IBS model (grey colon) groups before (**a**) and during intraluminal butyric acid (5 mM) infusion (**b**). *p < 0.05 compared to the control group

Analysis of the pellet transit rate in isolated colon demonstrated that in the mouse IBS model group it was significantly higher (22.11 ± 2.08%; n = 6, *p* < 0.05) compared to control (12.82 ± 0.59%; n = 6; Fig. 3a).

### Effect of SCFAs on contractility of colon segments

**Fig. 4:**
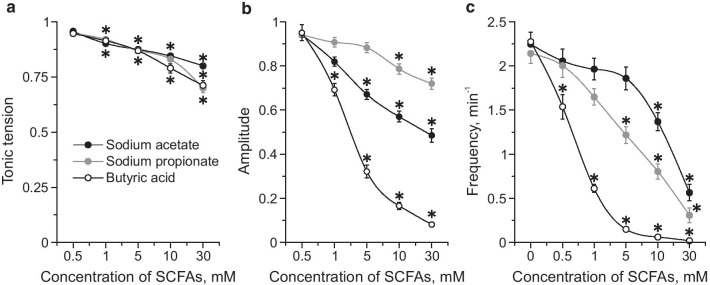
Dose-dependent effects of SCFAs on spontaneous contractions of isolated proximal colon. Effects of sodium acetate (black circles), sodium propionate (grey circles) and butyric acid (white circles) in concentrations of 0.5, 1, 5, 10 and 30 mM on tonic tension (**a**), amplitude (**b**) and frequency (**c**) of contractions of mouse proximal colon. * *p* < 0.05 compared to the initial value

**Fig. 5:**
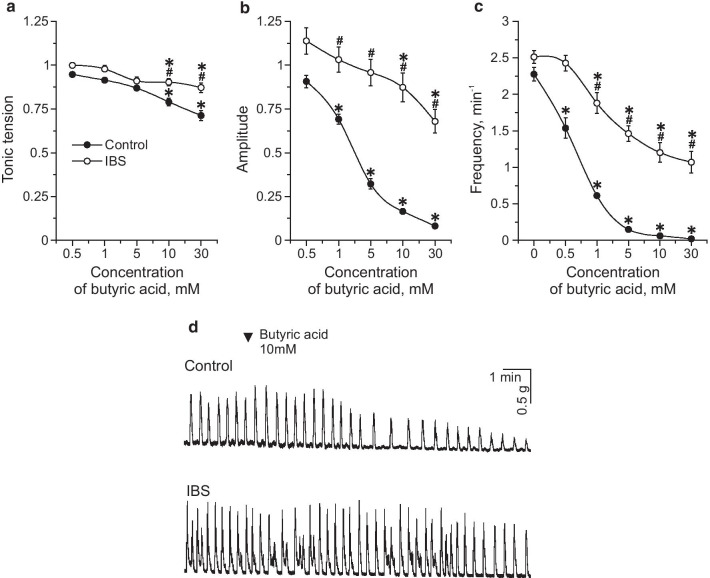
Effects of butyric acid on spontaneous contractions of mouse proximal colon in control and IBS model groups. Effects of butyric acid in concentrations of 0.5, 1, 5, 10 and 30 mM on tonic tension (**a**), amplitude (**b**) and frequency (**c**) of contractions of mouse colon segment of the control group (black circle) and in the IBS model group (white circle). **d** Original recordings of spontaneous contractions of proximal colon during butyric acid (10 mM) application in control and IBS model groups. *p < 0.05 compared to the initial value; # *p* < 0.05 compared to the effects of butyric acid in control.

To assess the effects of SCFAs on spontaneous contractions of the proximal colon, sodium acetate, sodium propionate and butyric acid were applied at concentrations of 0.5, 1, 5, 10, and 30 mM in IBS model and control groups. All SCFAs induced a dose-dependent decrease of tonic tension, amplitude and frequency of spontaneous contractions (Table 2; Fig. 4a–c). Butyric acid displayed the most pronounced inhibitory effect (Fig. 4a–c). EC_50_ of the inhibitory SCFAs effects on the amplitude of phasic contractions was 4.79 mM for sodium acetate, 7.58 mM for sodium propionate and 3.15 mM for butyric acid. In the IBS model group the inhibitory effects of all SCFAs on spontaneous activity were less profound compared to the control group (Table 3) which was particularly evident for butyric acid (Table 3; Fig. 5a–d).

**Table 2:** Contractile activity of mouse proximal colon during acute SCFAs application in the control group

Concentration of SCFAs (mM)	Tonic tension (g)	Amplitude (g)	Frequency (min^−1^)
Sodium acetate			
Initial value	0.781 ± 0.011	0.735 ± 0.037	2.5 ± 0.126
0.5	0.748 ± 0.012	0.677 ± 0.033	2.269 ± 0.116
1	0.704 ± 0.011*	0.584 ± 0.029*	1.965 ± 0.123*
5	0.681 ± 0.011*	0.489 ± 0.031*	1.86 ± 0.129*
10	0.659 ± 0.011*	0.423 ± 0.028*	1.368 ± 0.101*
30	0.621 ± 0.01*	0.378 ± 0.033*	0.565 ± 0.091*
Sodium propionate			
Initial value	0.84 ± 0.014	0.701 ± 0.028	2.14 ± 0.111
0.5	0.793 ± 0.013	0.662 ± 0.03	2.003 ± 0.132
1	0.771 ± 0.013*	0.631 ± 0.027	1.649 ± 0.095
5	0.73 ± 0.014*	0.613 ± 0.028*	1.218 ± 0.094*
10	0.695 ± 0.014*	0.547 ± 0.025*	0.805 ± 0.085*
30	0.591 ± 0.007*	0.497 ± 0.014*	0.308 ± 0.081*
Butyric acid			
Initial value	0.861 ± 0.008	1.097 ± 0.073	2.275 ± 0.091
0.5	0.815 ± 0.015	1.024 ± 0.077	1.537 ± 0.138
1	0.787 ± 0.015*	0.705 ± 0.037*	0.613 ± 0.044*
5	0.749 ± 0.015*	0.289 ± 0.02*	0.15 ± 0.032*
10	0.682 ± 0.02*	0.152 ± 0.009*	0.061 ± 0.025*
30	0.614 ± 0.026*	0.09 ± 0.006*	0.02 ± 0.01*

*p < 0.05 compared to the initial value

**Table 3:** Contractile activity of mouse proximal colon during acute SCFAs application in IBS model group

Concentration of SCFAs (mM)	Tonic tension (g)	Amplitude (g)	Frequency (min^−1^)
Sodium acetate			
Initial value	0.879 ± 0.01	0.884 ± 0.058	3.204 ± 0.117
0.5	0.85 ± 0.009	0.893 ± 0.024	2.694 ± 0.098*
1	0.791 ± 0.009*	0.801 ± 0.026*	2.643 ± 0.097*
5	0.864 ± 0.009*	0.783 ± 0.028*^#^	2.469 ± 0.121*
10	0.723 ± 0.008*	0.693 ± 0.029*^#^	1.857 ± 0.092*^#^
30	0.648 ± 0.007*	0.532 ± 0.034*^#^	1.143 ± 0.13*^#^
Sodium propionate			
Initial value	0.815 ± 0.021	0.848 ± 0.057	2.636 ± 0.102
0.5	0.814 ± 0.021	0.901 ± 0.026	2.33 ± 0.096
1	0.767 ± 0.02	0.795 ± 0.026	1.773 ± 0.08*
5	0.727 ± 0.019*	0.77 ± 0.029	1.417 ± 0.087*
10	0.698 ± 0.018*^#^	0.633 ± 0.027*^#^	1.045 ± 0.076*
30	0.516 ± 0.019*^#^	0.58 ± 0.029*^#^	0.693 ± 0.098*^#^
Butyric acid			
Initial value	0.855 ± 0.023	0.857 ± 0.046	2.512 ± 0.086
0.5	0.832 ± 0.021	0.873 ± 0.025	2.427 ± 0.107
1	0.811 ± 0.02	0.787 ± 0.023^#^	1.878 ± 0.143*^#^
5	0.757 ± 0.025	0.724 ± 0.026^#^	1.463 ± 0.105*^#^
10	0.772 ± 0.018*^#^	0.652 ± 0.032*^#^	1.203 ± 0.133*^#^
30	0.714 ± 0.021*^#^	0.501 ± 0.033*^#^	1.069 ± 0.147*^#^

*p < 0.05 compared to the initial value; ^#^*p* < 0.05 compared to the control group

Incubation of proximal colon specimen in sodium acetate, sodium propionate or butyric acid at a concentration of 10 mM decreased the amplitude and AUC of carbachol-induced contractions in the control group (Fig. 2a–c). In IBS model group the inhibitory effects of sodium acetate, sodium propionate were non-significant, whereas incubation in butyric acid decreased carbachol-evoked contractions, but its effect was lower than in the control group (Fig. 2a–c).

Luminal infusion of butyrate, in a concentration of 5 mM, into isolated colon accelerated artificial pellet propulsion in both groups. In control colonic transit was 24.33 ± 1.56% (n = 6) and in the mouse IBS model group significantly higher—29.95 ± 0.72% (n = 6, *p* < 0.05; Fig. 3b).

### SCFAs in feces assessed by gas–liquid chromatography

**Fig. 6:**
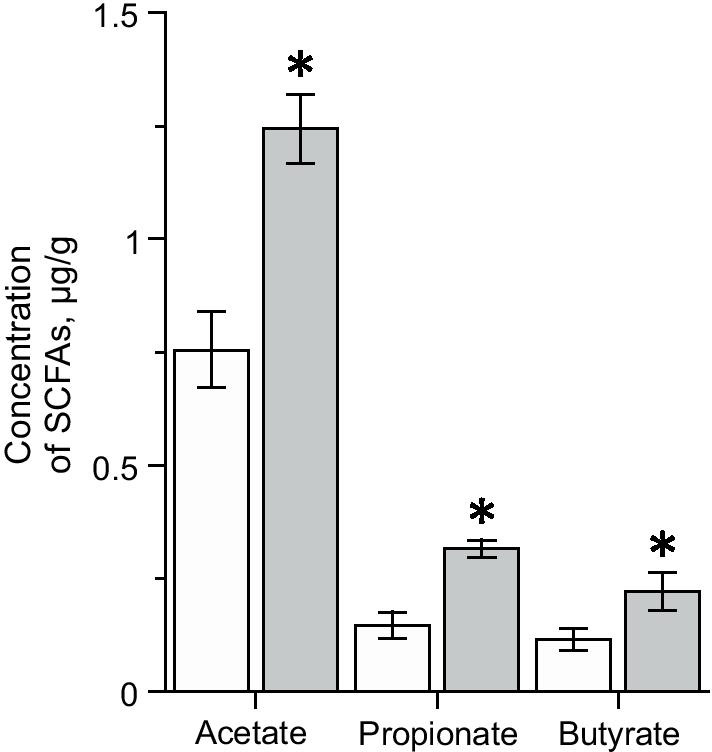
Concentration of acetic, propionic and butyric acids in the feces of control (white column) and IBS groups (grey column) of mice. *p < 0.05 compared to control

In control mice the ratio of acetate (0.75 ± 0.07 µg/g, n = 5), propionate (0.15 ± 0.03 µg/g, n = 5) and butyrate (0.11 ± 0.02 µg/g, n = 5) was 6.5: 1.3: 1 (Fig. 6). In the mouse IBS model group the levels of all SCFAs were significantly higher compared to the control group: acetate concentration increased by 65% (n = 5, *p* < 0.05), propionate—by 114% (n = 5, *p* < 0.05) and butyrate—by 91% (n = 5, *p* < 0.05) (Fig. 6). The ratio of acetate: propionate: butyrate changed to 5.6: 1.42: 1. The overall level of volatile fatty acids in the IBS group increased from 1.115 ± 0.124 µg/g in the control group to 1.907 ± 0.093 µg/g (n = 5, *p* < 0.05).

## Discussion

Irritable bowel syndrome (IBS) is a functional bowel disease in which abdominal pain is associated with impaired colon motility and visceral hypersensitivity. Alteration in gut microbiota and its metabolites appear to promote development and maintenance of IBS symptoms [7]. It was proposed that bacterial fermentation is impaired in IBS with subsequent alteration of the intestine motility pattern [23], however, the effects of SCFAs on colon motility in an IBS model were not investigated.

SCFAs are the end-products of anaerobic colonic bacterial fermentation of nondigestible carbohydrates or proteins proposed in the pathogenesis of IBS, including their effects on intestinal motility [23]. We analyzed the concentration of SCFAs in the feces of mice using an IBS model. Our data show that the overall concentration of volatile fatty acids was elevated in IBS which agrees with human studies of fecal SCFAs content in IBS-D (patients with predominant diarrhea) [37].

Moreover, it was shown that IBS-D and IBS-C patients exhibited different profiles of SCFAs [6]. Recent meta- analysis demonstrated the increased level of propionate and butyrate in IBS-D with a fast transit in feces [24, 38], which is consistent with our finding of relative abundance of propionate and butyrate levels compared to acetate in the mouse IBS model.

SCFA levels in feces depend not only on the microbiota content, but also on the colonic absorbance and transit time [39, 40]. Indeed, it was shown that intraluminal SCFAs are very efficiently absorbed in the colon, and only 5 to 10% of the SCFAs produced by bacterial fermentation are excreted and can be measured in the feces [39, 40]. However, altered SCFA levels in feces are accompanied by changes in microbiota with relative abundance of SCFA-producers, such as *Clostridia*, *Bifidobacteria, Ruminococccaceae* and *Erysipelotrichaceae* families [41]. A  different distribution of *Clostridiales* was shown in patients suffering from IBS-C or IBS-D [38]. Therefore, as products of gut microbiota, SCFA level reflects the status of the microbiota. Moreover, in a recent study increased levels of propionate and butyrate in IBS-D in blood serum were discovered which corresponds to our data [42]. Hence, although it has been reported that fecal and mucosal microbiota are structurally diverse they are highly correlated [43], and can be used to discriminate between IBS-D subjects and healthy controls [44].

Next, we compared the colon transit rate and parameters of spontaneous and carbachol-evoked contractions of the proximal colon in control and in the IBS model. Animals from the IBS group exhibit visceral hypersensitivity, which along with smooth muscle hypercontractility were proposed to be responsible for abdominal pain [1]. The model of post-inflammatory IBS used in our study is considered to be a model of IBS-D [45, 46] where colonic transit is generally accelerated compared to IBS-C with slow gut transit [1, 47, 48]. Indeed, in our model of IBS we found acceleration of the colon transit which was accompanied by an increased amplitude and frequency of spontaneous contractions of proximal colon preparations, similar to previous data by Jia et al. [49]. Cellular mechanisms responsible for increased colonic motility in IBS may include up-regulation of L-type calcium channels in colonic smooth muscle and/or an increased number of interstitial cells of Cajal (ICC) due to the expression of 5-HT2B receptors [25, 50]. Colonic hypermotility may result not only from myogenic changes, but also involve potential changes of the enteric nervous system and brain-gut axis signaling [51]. Chronic elevation of SCFA level in IBS-D, especially butyrate was shown to increase the numbers of cholinergic and nitrergic neurons which are key excitatory and inhibitory motor neurons mediating the peristaltic reflex and propulsion [14, 52]. Indeed, carbachol-evoked contractions of the colonic segment in our experiments were reduced in the IBS model group which may reflect alteration of cholinergic mechanisms at the level of smooth muscle or neuronal cells. Additionally, an elevated number of enterochromaffin cells and a higher percentage of degranulated mast cells along with elevated levels of 5-HT were shown in patients with IBS-D. 5-HT in turn initiates high-amplitude, propagated colonic contractions, accelerated intestinal transit, and increased gut motility [53–55]. Thus, we suggest that increased motility and shorter transit times are associated with elevated endogenous levels of SCFAs in the IBS mouse model.

We further compared the acute effects of exogenous SCFAs on colonic transit rate and spontaneous and carbachol-evoked contractions of proximal colon specimen in control and IBS mouse model groups. Under physiological conditions, SCFA levels in peripheral blood are very low (in the µM to low mM range) due to hepatic metabolism, with acetate being the main SCFA in circulation [56, 57]. In our study, we used millimolar concentrations of SCFAs which are corresponding to the luminal level of SCFAs in vivo [12]. In control groups, SCFAs dose-dependently decreased tonic tension, amplitude and the frequency of spontaneous contractions of proximal colon, whereas in the IBS model group these inhibitory effects were less pronounced. Similar, carbachol-evoked responses were decreased after incubation of colonic preparations  in SCFAs in control, but they were less sensitive to SCFAs in the IBS model group.

Inhibitory action of SCFAs on motor activity of proximal and distal rat colon specimens was shown previously [15, 16, 18, 58]. However,  at low concentration SCFAs produced contractile responses and only in mucosa attached preparations [20, 22, 52, 59]. Moreover, SCFAs accelerated colonic transit during intraluminal administration [14, 54]. Indeed, in our experiments intraluminal infusion of butyrate increased colonic transit in both groups, with a larger effect in the IBS model group. Similarly, in the proximal colon of the guinea pig butyrate increased propagating but decreased non-propagating contractions which were proposed to accelerate transit [13].

The stimulatory effect of SCFAs on colonic transit was suggested to be mediated through release of 5-HT from enteroendocrine cells which initiates or augments the peristaltic reflex [54, 60]. The inhibitory effect of SCFAs appears caused through the inhibitory action on nicotinic ACh receptors of cholinergic nerve terminals [18] and release of peptide YY (PYY) from enteroendocrine cells, which inhibits colonic propulsive motility [17].

Activation of Free Fatty Acid receptor (FFA3), expressed in presynaptic cholinergic terminals and receptors FFA2/FFA3 in enteroendocrine cells releasing PYY and Glucagon-Like Peptide-1 (GLP-1) are considered to be involved in the motility effects of SCFAs [19, 61, 62]. However, only inhibitory action on gut motility and mouse colonic transit rate has been observed in vitro using potentially selective FFA2 and FFA3 agonists [32, 63, 64]. Moreover, FFA3-deficient mice exhibited accelerated intestinal transit and low plasma concentrations of PYY [62]. Indeed, FFA2 and FFA3 expressed in enteroendocrine cells are co-localized with PYY but not with 5-HT receptors [19, 21]. Therefore, the source of 5-HT released by SCFAs might be FFA2-immunoreactive mast cells [19]. At the same time, antagonists of FFA3 did not prevent the release of PYY from enteroendocrine cells by SCFAs [65] which suggest that at least part of the SCFA effects are not dependent on the activation of FFA receptors. Indeed, SCFAs can directly change excitability of neuronal or smooth muscle cells. Thus, butyrate was shown to hyperpolarize cultured myenteric neurons through an increase of intracellular Ca^2+^ followed by activation of K^+^ channels [66, 67] and acetate induced hyperpolarization by direct activation of big-conductance Ca^2+^-activated K^+^ channels [68, 69].

## Conclusion

In conclusion, our results indicate that in mouse IBS model the chronically elevated SCFA level is associated with an accelerated colonic transit and an increased amplitude and frequency of contractions of the proximal colon specimen. SCFAs induced an inhibitory action on colon segment contractility, whereas intraluminal butyrate administration increased colonic transit. In the mouse IBS model inhibitory effects of SCFAs were less pronounced, however, the stimulatory effect was stronger compared to control. We suggest that motor and neuronal mechanisms of contractility regulation in IBS became insensitive to the inhibitory action of SCFAs, whereas stimulatory effects through colon mucosa became overexpressed. Therefore, elevated SCFA levels in the IBS impairs the balance between stimulatory and inhibitory effects of SCFAs on colon motility which results in an accelerated transit in IBS with predominant diarrhea syndrome with hyperexcitability and hypermotility.

## Data Availability

The datasets during and/or analyzed during the current study are available from the corresponding author on reasonable request.

## Abbreviations

AUC: Area under the curve
AWR: Abdominal withdrawal reflex
IBS: Irritable bowel syndrome
SCFAs: Short chain fatty acids
IBS-C: IBS with predominant constipation
IBS-D: IBS with predominant diarrhea
IBS-M: IBS with mixed bowel habits
